# Indignity of Nazi data: reflections on the utilization of illicit research

**DOI:** 10.1007/s11019-024-10212-z

**Published:** 2024-06-06

**Authors:** Iman Farahani, Joel Janhonen

**Affiliations:** 1 Institute of Biomedicine, and MediCity Research Laboratories, University of Turku, Turku, Finland; 2 Turku Bioscience Centre, University of Turku and Åbo Akademi University, Turku, Finland; 3 Faculty of Medicine, University of Turku, Turku, Finland; 4 The Finnish Institute of Bioethics, Tampere, Finland

**Keywords:** Medical research, Research ethics, Human dignity, Moral complicity, Unethical human experimentation

## Abstract

Human rights may feel self-apparent to us, but less than 80 years ago, one of the most advanced countries at the time acted based on an utterly contrary ideology. The view of social Darwinism that abandoned the idea of the intrinsic value of human lives instead argued that oppression of the inferior is not only inevitable but desirable. One of the many catastrophic outcomes is the medical data obtained from inhuman experiments at concentration camps. Ethical uncertainty over whether the resulting insights should be a part of the medical literature provides a chance to consider the seemingly irreplaceable social construct of human dignity. Would any medical benefit justify the utilization of this illicit data? Would utilization even qualify as an insult to the dignity of the exploited subjects, or is this a question about intersubjective meaning? This work discusses the wisdom in blind adherence to human dignity, the possibility of retrospective insults, moral complicity, contrary viewpoints, and possible resolutions.

## Introduction

In biomedical ethics, a nuanced and complex question arises regarding the ethical utilization of scientific data derived from unquestionably unethical research. This ethical conundrum, though seemingly straightforward at first glance, unveils layers of intricacy upon closer examination. The issue gained historical prominence, particularly in the aftermath of World War II, when revelations about the scientific experiments conducted under the Nazi regime came to light. This period saw an intensified scrutiny of the ethical implications surrounding the use of data emanating from morally reprehensible research practices. In the pursuit of a systematic exploration of this moral dilemma, this inquiry centers on the examination of Nazi medical research data. By delving into this historical context, we aim to unravel the complexities inherent in the ethical considerations surrounding the utilization of scientific findings derived from studies tainted by ethical transgressions and violations against human rights.

Regrettably, during the era of the Nazi regime, a dark departure from present-day ethical standards occurred as Jewish individuals were subjected to egregious violations of their autonomy, standing in appalling juxtaposition to the conventional use of animals in research. Nazi researchers conducted experiments on the Jews as human models to gain data on life-threatening conditions such as hypothermia or hypoxia, as well as monitored disease progression by infecting Jewish children (Nazi Medical Experiments 2006). While these instances are merely a fraction of the brutal experiments carried out by Nazi researchers on human subjects, they suffice to illustrate the gravity of the ethical transgressions for the purpose of this exploration. Certain aspects of Nazi racial ideology, such as racial inequality, the imperative nature of the racial struggle for existence, and collectivism, were fundamentally rooted in Darwinian theory (Weikart 2013). The underlying philosophy of social Darwinism argues that the value of people can be functionally determined. Thus, instead of considering everyone to have inherent human dignity constant competition and progress should be embraced, and the powerful should not feel sorry for the lesser (O’Mathúna 2006). Based on perceived racial inferiority, Jewish lives were considered expendable for scientific ends.

Despite social Darwinists considering ethical considerations merely as undue barriers to natural competition, it is crucial to reassess the moral standing of these experiments in the present-day normative context. This reassessment involves evaluating experiments for potential violations of the four principles of biomedical ethics. Non-maleficence, to not harm the patients, was violated by causing injury and death to the participants with no intention to benefit them. Beneficence, considering the welfare of the research participants, was violated due to the nature of the experiments. The principle of autonomy was broken since the prisoners had no consent to engage in this research and were not allowed to make an informed decision on their participation. Finally, the principles of distributive justice, treating all people the same in medical care and medical experiments regardless of gender, age, ethnicity, religion, and social status, were violated since particular races were targeted with genocidal intentions (Beauchamp and Childress 2001).

In the aftermath of World War II, certain studies conducted during that tumultuous time found their way into scientific research literature. Now, as humanity has overcome its darkest era, we are well aware that even doctors can perform evil actions in search of knowledge. Yet, lingering questions persist regarding the ethical use of this data. Do we harness it for the betterment of humanity, leveraging it for medical advancements? Or do we grapple with the moral dilemma of burying it, as it bears the scars of Nazi atrocities? In essence, can we ethically glean knowledge from data born out of personal and societal suffering, all in pursuit of medical progress?

Before exploring this ethical question, we should suppose that data stemming from some Nazi medical studies are valuable and could benefit today’s medical research. However, the validity of many of these studies is in question, and the methodologies have often been criticized for being unscientific and flawed (Halpin 2008). Moreover, the inability to replicate Nazi experiments calls into question their scientific validity. Because it is ethically unthinkable to reproduce their results, their validity remains in question prior to utilization and possible breakthroughs. This precludes a standard assessment of their reliability and limits their use in contemporary scientific discourse. Nevertheless, to lay out all ethical considerations and trade-offs, we might even suppose that the data contains truly unique scientific insights that promise to advance medical research and possibly save lives and alleviate suffering.

In this work, we explore this problem by reviewing for-and-against arguments separately. We believe that using Nazi research data under certain circumstances can be ethically sound, as explained in the conclusion section with a proposed tentative solution.

## Arguments in favor of using the data

Typical utilitarian and pragmatic viewpoints would argue in favor of using Nazi research data (Wilson 2011). Utilitarianism is exemplified in Jeremy Bentham’s “*Introduction to Morals and Legislation*.” Bentham posits that the moral worth of action should be assessed regarding human happiness, advocating for the pursuit of “the greatest happiness for the greatest number” (Veenhoven 2010). Notably, a utilitarian would not defend the Nazi methodology since the unethical experiments barely provide the greatest happiness or well-being for the greatest number. This sort of research would seriously damage the credibility of the medical profession and so have severe and far-reaching negative consequences (Niburski 2014). Utilitarians generally recognize that even if the benefit would exceed the harm in a single instance, the overall consequences may be catastrophic if the same decision is normalized as a rule. However, since the harm has already been done and no one can undo the past, using these data may save lives, avoid life-threatening conditions, and treat diseases, leading to greater well-being for more people. Also, utilitarians see no real difference between acts and omissions, so anyone discarding possible medical insights would bring about further suffering. The moral paralysis over the first travesty would give rise to a second unnecessary loss. This is the most substantial reason for using unethically obtained data in the current scientific investigations.

From a pragmatic perspective, it is possible to argue that data such as numbers, by their nature, are morally neutral and cannot be called good or bad. Would it not be futile or even deranging to vilify a dataset and consider it ‘untouchable’? It would follow that research material may be used regardless of whether it is obtained ethically. The origin does not matter; what can be achieved here and now matters. Behind this superficially reasonable argument, a straw man fallacy is concealed. One should consider that what is deemed unethical is ‘using the data obtained unethically’, not the ‘data’ itself. The complexity of the natural language makes one refer to it as ‘unethical or illicit data’, which logically means the data acquired with unethical approaches. So, being unethical is not implied to the data in the first place but using unethically obtained data. Above all, ethical pragmatists are concerned about the usefulness of things, behaviors, and social constructs. Therefore, if it turns out that human dignity is an irreplaceable construct for the functioning of society, the mental linking of Nazi data to evil could be a very useful mental habit indeed.

Arthur L. Caplan contends that the widespread dissemination of Nazi data is crucial for condemning the immoral biomedical research conducted during that dark period. Moreover, his perspective argues in favor of using this data if it can save lives, underscoring a utilitarian approach. In alignment with Caplan’s viewpoint, Velvl W. Greene advocates against the omission and destruction of Nazi data, emphasizing the importance of preserving historical records, even those associated with heinous acts, to prevent a slippery slope of data suppression and destruction. Much of early medical knowledge would not survive if all that undermines self-determination or reminds us of past indignities would be deemed illicit. Both Caplan and Greene, albeit from different angles, lend their voices to the idea of preserving Nazi data as a means of establishing a historical exemplar of wrongdoing. However, Caplan underlines clear moral denunciation of how the data was obtained (Caplan 1992; Jacobi 2017). Moreover, he emphasizes being careful and respectful in choosing our language, as the normative significance of our descriptors can impact the perception of events and the legitimacy conferred upon them, especially over the use of Nazi data (Caplan 2021).

One may argue that using this data in current research for the good of humanity gives ‘value and meaning’ to those who have lost their lives in these unethical experiments. By focusing on the instrumental value of these lives, this sentiment echoes the unfortunate idea that the value of these people is based on their function as research subjects. Rather than acknowledging the dignity of each person, the status of these lives is considered lesser and contingent on their worth to others. The cynicism in this argument presumes that the value of a human being is primarily a relational construct dependent upon conditions such as perceived social utility or racial status. In contrast, this is generally recognized as an inherent quality of being human, independent of both the Darwinian considerations and factors above.

In turn, ‘meaning’ is not simply established by actions or their material effects – nor is it assigned at will. Instead, it is subjectively interpreted and intersubjectively negotiated. The meaning-making process is much less concerned with prospective research findings than with recognitions of significance, intentions, and satisfying resolutions. In his book “*Man’s Search for Meaning*,” Viktor Frankl emphasizes the subjective nature of meaning by stating, “Everything can be taken from a man but one thing: the last of the human freedoms—to choose one’s attitude in any given set of circumstances, to choose one’s own way” (Frankl 1959). This discovered sense of agency to imagine the purpose amid suffering helped Frankl and many others during their imprisonment at Nazi concentration camps. Perhaps the enduring hardship caused by instances of illicit experimentation could be mitigated by a joint search for meaning among stakeholders.

## Arguments against using the data

One crucial question is about the ethical consequences of utilizing illicit data for the person conducting such secondary research. Thomas Aquinas’s nine principles of moral complicity offer valuable insights here (Mellema 2008). In this instance, the moral ‘complicity of flattery’ becomes relevant, as the use of Nazi research data essentially endorses the entirety of the research, including its methodology, although inadvertently. In case the reprehensible means by which the data was obtained are not denounced, there is also implication in the moral ‘complicity of not denouncing’. For a utilitarian, complicity in moral issues is not applied if one’s action does not affect the consequence. The justice system, in turn, only recognizes an even narrower notion of complicity; being an accessory to a crime requires aiding or abetting (Mellema 2011). Moral complicity could, however, include additional forms of unethical involvement other than what can be practically enforced. The idea that Nazi doctors and inhuman experimentation advanced someone’s research interests can surely create a morally distasteful association and so impact the perceived meaning of such work – even if it would be done with the best of intentions.

The idea that we should avoid any cooperation with evil and so not become complicit in it – is especially widespread in Catholic ethics. Besides not committing illicit actions, people should retain a remoteness to them. Moral complicity is considered to exist in different forms and degrees. For example, medical research on certain embryonic cell lines would qualify as cooperation in evil – or the connected practice of abortion. Instead, taking a resulting COVID-19 vaccine was recently considered sufficiently remote and acceptable as only a form of passive material cooperation (*Congregation for the Doctrine of the Faith*, 2020). Whether agreeing with the Vatican on matters like abortion or not, these differing degrees of remoteness and moral complicity could be useful when applied to the present case. Any patient that would receive medical benefits from this research would not have actively collaborated with the Nazis. However, knowingly selecting this dataset of illicit origin for research would likely qualify as active material cooperation with Nazi human experimentation. A researcher would not, of course, be guilty of infecting children, and there would be some distance to such evil actions, yet a free choice was made to stand and build upon them.

Furthermore, from the rule-utilitarian perspective, which considers the consequences of collective actions rather than their moral complicity, the utilization of Nazi data may convey a message to upcoming scientists that they can bargain with malevolence and exploit it, prioritizing scientific advancements over human dignity. Hence, this approach would not guarantee the attainment of the greatest good for the greatest number since adopting this idea would destroy the moral standing and credibility of the scientific community and end up exaggerating human suffering (Vigorito 1992; Wilson 2011). Adopting a general rule to avoid cooperation in what one understands as evil would, in turn, likely make the world all-in-all a better place.

On a related note, some deontological ethical theories would dispute using Nazi research data. From this perspective, no one should ever violate sacred duties toward our fellow humans to accomplish anything; the end does not justify the mean. For example, Kantian ethics mostly cares about rules and motives, whereas utilitarianism, or any consequentialist ethic, only considers the outcome (Wilkens 2011). In this respect, Kantian ethics were violated in two ways. First, Nazis treated people like ‘things’, not ‘human beings’, so they hurt them intending to obtain scientific data for the good of a particular group of people. By extension, using such research material to pursue further scientific or medical ends undermines the self-determination and dignity of the ‘human means’. The consent remains unasked and unreceived. Such secondary research would arguably perceive the victims as not yet fully tapped-out resources waiting to be exploited. Second, if one believes utilizing Nazi data to be unethical, conducting such research, with any good intention, say, healing the sick, can still be counted as a form of Kantian ethics violation. Neither good outcomes nor intentions can undercut our obligations.

From the Kantian standpoint, dignity is viewed as an intrinsic and unconditional value, existing independently of functionality or contextual factors. Consequently, the inhumane experiments conducted by the Nazis cannot affect the dignity of the victim. However, Quinn emphasizes that Kant’s perspective fails to adequately address the survivors’ claims of harm. Instead, Quinn proposes an alternative conception of dignity that better aligns with the experiences of survivors. Quinn seeks to substantiate the survivors’ claims by associating dignity with control, specifically self-determination, and acknowledging that individuals exercise varying degrees of control and, hence, possess varying degrees of dignity. According to this framework, victims lost a significant amount, if not all, of their dignity during the Nazi experiments. Quinn suggests that victims, or their family members and surrogates, must maintain control over the data. This control becomes instrumental in restoring their dignity, even posthumously, as it ensures the continuation of their projects. Without such control, their dignity remains at risk over the usage of such data (Quinn 2018). Quinn’s view has hopeful implications, as the meaning of these victimized lives is not ultimately settled by the actions of Nazis; instead, their meaning can be renegotiated and their lost dignity can be restored by those caring for them in the present.

## Lessons to be learned for today

From a broader perspective, it is crucial to be cautious with utilitarian thinking – the idea that the end justifies the means for the greater good. A caricature version of the act-utilitarian approach can appear to justify actions that raise complex ethical concerns. Fyodor Dostoevsky’s novel “*Crime and Punishment*” illustrates this through its protagonist, Raskolnikov, who justifies murdering a pawnbroker on utilitarian grounds, claiming that a ‘louse’ has been removed from society – motivated by a belief in his right to commit a morally ambiguous act for ‘the greater good’. Yet, as the narrative unfolds, his actions lead to a torment of guilt and psychological turmoil, revealing the moral and existential crises stemming from his utilitarian reasoning. Dostoevsky’s work warns against oversimplifying moral dilemmas and emphasizes the significant impact of utilitarian ethical approaches on individuals and society.

Moreover, the basic notion of life’s inherent dignity connects to many bioethical issues worthy of elaborated and ongoing debates, including the conditions necessary for full moral status or personhood. The conversation should not end with a simple notion of human dignity; setting the threshold for moral consideration to ‘humanness’ may only be useful up to a point. From an alternative view, this is mere speciesism or the undue prioritization of our interests above non-human animals. Strict adherence to the notion of human dignity can even contribute to the exploitation of animals (Singer 1990). But, as a starting point for both scientific and medical research, moral wariness about destroying or using human life seems sensible and historically well-justified.

When considering the utilization of Nazi research data, the complications intensify if we push the utilitarian approach to its extremes. Suppose within this controversial data lies potentially groundbreaking information on cancer treatment, surpassing current research advancements. In such a scenario, opting for a utilitarian approach might be deemed a well-justified choice for those earnestly seeking viable treatment options. Given that the ethical intuitions can flip at some point when the outcomes are exacerbated, where does the burden of proof lie? On those adhering to a strict notion of human dignity? Or instead on utilitarians to prove that somewhat undermining this social construct constitutes the lesser evil in a particular case?

## What to do

As discussed, the final call on using Nazi research data continues to divide opinions and challenge preconceptions. As an example, one prominent voice in this debate, Dr. Robert Pozos, initially used and argued for the utilization of this material. However, by 1987, he started questioning the ethical implications of such usage. Ultimately, in 1992, he concluded that Nazi data should not be cited, i.e., acknowledged, but still made available to scientists to advance humankind’s understanding. He recommended that every researcher individually determine their stance on the matter, and personally, he opted against incorporating controversial data into his work (Jacobi 2017). Instead of leaving individuals to decide for themselves, we advocate for implementing a structured framework that encourages deliberation and meaning-making across concerned parties.

We hold that using Nazi research data can be permitted under certain circumstances. For instance, the use of data should be limited according to the importance of the research. The research aims should be substantial and beneficial for life-threatening diseases or conditions, e.g., these data should not be used to develop cosmetic products or associated with lower priority studies. To objectively evaluate and prioritize health outcomes, Quality-Adjusted Life Years (QALYs) serve as a pivotal metric. QALYs offer a comprehensive measure that considers both the quantity and quality of life, providing a standardized framework for gauging the influence of a healthcare intervention on an individual’s overall well-being (Whitehead and Ali 2010). Establishing an enforced threshold through a minimum expectation of QALYs provides a standardized means for objectively evaluating the implications of using such data. A rigorous evaluation would emphasize the seriousness of utilizing these records and the moral gravity surrounding them. Besides an objective metric, the inter-subjective factors should not be discounted.

We propose the establishment of an institute under UNESCO’s Social and Human Sciences (SHS) sector to centralize and manage all unethical yet scientifically valuable data, creating a distinct registry of cases of unethical research. Utilization of these data would be permissible only upon obtaining approval from a board composed of bioethics experts, physicians, biomedical specialists, and representatives of the victims. A procedure should be created that allows local research ethics committees to contact the founded UNESCO authority regarding proposals to utilize illicit data, initiating a review and enabling broader deliberation. Rather than just checking if a request fulfills a set of to-be-determined criteria, the institute should facilitate discussions on dignity and possible ways to restore it. For this to become standard practice, initial reviews by the local research committees and editorial boards should actively flag and problematize unapproved secondary research.

Additionally, to acknowledge the special ethical gravity of one’s research and demonstrate appropriate care when investigating such data, all references to these data should be without the names of original researchers and noticeably marked with specific indicators, signaling them as unethical research. Moreover, authors analyzing and citing such data should be encouraged to pledge appropriate resources of time and money to support the efforts of the institute. These could fund the review process and be generally allocated to enhance public awareness of medical ethics through various initiatives. This model could extend beyond Nazi experiments to cover diverse instances of unethical research, conceivably including morally abhorrent cases of animal experimentation.

In his book “*Means, Ends, and Persons*” Robert Audi provides a valuable tool to assess the moral content of the provided solution by introducing three dimensions: act type, motivation, and manner of performance (Audi 2016). Accordingly, whether or not human dignity is undermined follows not only from what is done but what is intended and how exactly this intention is carried out. The motivation behind the presented solution is maintained to be morally adequate because it solely focuses on improving essential health outcomes while also preserving the dignity of the victims. Furthermore, the manner of performance is also considered adequate because the data is used without disclosing the identities of the original researchers and is clearly marked to have an unethical origin. Additionally, the idea to pledge resources seeks to increase beneficence by facilitating awareness raising and ethical deliberation.

The objective of this work is not to lay out a comprehensive proposal but to introduce an idea of a negotiative oversight procedure for the utilization of illicit data. We propose this to spark further consideration and debate among those with legislative and institutional expertise. While we have suggested some initial criteria for assessing applications, this preliminary framework needs elaboration and refinement. We hope that others will contribute additional viewpoints for consideration and help develop a comprehensive set of criteria to forward its implementation.

The choice between either undermining human dignity or neglecting the needs of the living is a false one. In contrast to what has been argued, we do not need to anchor biomedical research to strict Kantian obligations to ethically deal with products of prior research (Niburski 2014). People who are waiting for new interventions are nevertheless likely to value similar things to people victimized by unethical research. For each aftermath of illicit human experimentation, many different stakeholders should be involved and listened to. A morally sensitive research framework allows individuals and groups to seek resolution and restoration of dignity in different ways. This is a very human process and indescribable by abstract or absolute normative ideals.

## Conclusion

Even though many decades have elapsed since the heinous Nazi experiments on humans, the ethical dilemma surrounding the utilization of the data continues to be a subject of active discourse, with no definitive resolution in sight. Nonetheless, we find solace in residing in a world where the ethicality of using such data is debated and scrutinized from many insightful perspectives. Debating such questions can elevate society’s moral awareness, even without ever providing final answers. Nevertheless, from the viewpoint of an individual researcher, there are non-negotiable principles and guidelines that must be adhered to in one’s studies. Although imperfect, these constructs and norms are a valuable achievement of humanity.

Broader social outcomes largely depend on the credible recognition of dignity. Even perceived insults to self-determination or the construct of inherent human value can undermine social cohesion and the critical collective commitment to human equality. Well-founded adherence to constructs like human rights and dignity makes us stronger together. But instead of blindly sticking to these notions and never questioning them, these need to be continually deliberated, renegotiated, and revised to remain relevant and in effect.

Humans are endlessly inventive in imbuing senseless past tragedies with meaning and significance. An appropriate moral framing of the use of Nazi data acknowledges the unique value of each life lost, so refuting their expendability. Only with this recognition can any illicit data find a meaningful place in the body of scientific knowledge. If, despite the genocidal intentions of the experimenters, something valuable can be discovered to help all humankind – including those assigned for destruction – this process could be seen in terms of moral resolution and amelioration. The dignity of individuals who were abused in these experiments is jeopardized only if the violations against their self-determination are considered not to matter.
